# Partial Synthesis of Crassicauline A from Yunaconitine

**DOI:** 10.1007/s13659-020-00238-0

**Published:** 2020-04-15

**Authors:** Rong-Ping Zhang, Yan-Jun Lin, Hao-Fei Yu, Si-Ying Chen, Jun Zhou

**Affiliations:** 1 Yunnan University of Chinese Medicine, Kunming, China; 2 Kunming Medical University, Kunming, China; 3 Kunming Institute of Botany, Chinese Academy of Sciences, Kunming, China

**Keywords:** Diterpenoid alkaloids, Yunaconitine, Crassicauline A

## Abstract

**Abstract:**

Both *Aconitum hemsleyanum* and *Aconitum geniculatun* have abundant contents of yunaconitine **(1)**. Yunaconitine **(1)** has similar skeleton to crassicauline A **(3)**; the only difference between them is that **1** contains a α-hydroxyl group at C-3. Our team attempts to convert **1** into **3** because **3** owns pharmacological activity. There are two steps to achieve the transformation above: firstly, use dehydration reaction to transform yunaconitine **(1)** into dehydroyunaconitine **(2)**; secondly, use hydrogen reduction to acquire crassicauline A **(3)**. Compared with other methods, this one below is more suitable for production application and more concise; moreover, the cost is lower with higher yield.

**Graphic Abstract:**

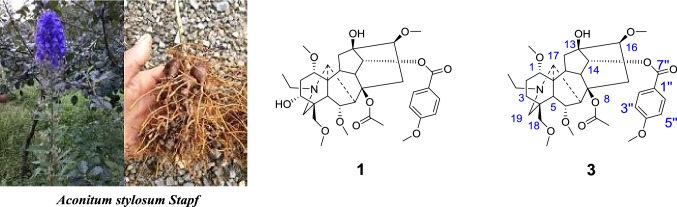

## Introduction

Diterpenoid alkaloids are the main pharmacological constituents of *Aconitum*. Diterpenoid alkaloids have anti-inflammatory, analgesic, sedative, antipyretic, and antineoplastic effection; the weight of them is accounting for about 7%–10% of *Aconitum*. Yunaconitine **(1)**, as a C-19 diester diterpene alkaloid, distributes in almost the whole Yunnan Province; *Aconitum hemsleyanum* and *A. geniculatum* contains yunaconitine **(1)** [1–3] abundantly. However, **1** has very strong toxicity (LD_50_ for mice is 585 μg/kg (i.p.), for rats is 50 μg/kg (i.v.), and for dogs is 30 μg/kg (i.v.) [4].). Additionally, although crassicauline A **(3)** has high treatment index, low toxicity, strong analgesic activity and no pain tolerance, it is lack of contents in *Aconitum* [5–9]. Moreover **3** [10] exists in a tiny minority of *Acontium s*pecies that produced in Li Jiang, Yunnan, China. Hence, there is a high cost in the application of **3**. At present, **3** has been widely used in clinical treatment for more than 30 years in China [4] including the treatment of rheumatoid arthritis, osteoarthritis, neuropathic and chronic pain. The main available dosage forms of crassicauline A **(3)** on the market include tablets, capsules, injections. Some studies have shown the subtle difference between **1** and **3**: Yunaconitine **(1)** contains a α-hydroxyl group at C-3; we believe that partial synthesis of **3** from **1** deserves to be mentioned.

A patent published by Zhang et al. [3] has reported four partial synthesis methods to afford crassicauline A **(3)** but the methods are complicated and difficult to purify the products with low yield. Therefore, we try to obtain semi synthetic **3** on the basis of increasing yield and getting a concise route.

## Results, Discussion and Conclusion

**Fig. 1:**
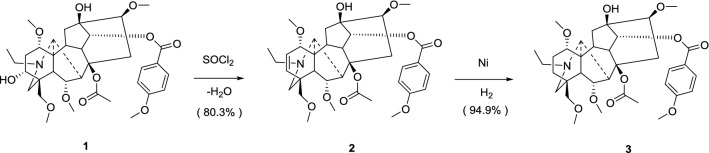
Chemical transformation of **1** to **3**

At first, yunaconitine **(1)** was treated with thionyl chloride to acquire a dehydration product. Then the product was treated with silica gel column chromatography. After that, dehydroyunaconitine **(2)** (Fig. 1) was obtained. The ^1^H NMR spectra (*δ*_H_ 6.01, 5.77) and ^13^C NMR spectra (*δ*_C_ 137.6, 125.3) of **2** illustrated the presence of a double bond at C-2. Besides, in HRESIMS^+^, a quasi-molecular ion peak appeared at *m/z* 642 [M+H]^+^; we could totally confirmed the double bond again. Lastly, under hydrogen reduction with Raney Ni catalysis, dehydroyunaconitine **(2)** converted to another product **3** (Fig. 1) which showed the same data (^1^H NMR, ^13^C NMR, ESIMS) as standard sample of crassicauline A. In this way, semi synthesis of crassicauline A **(3)** from yunaconitine **(1)** had been completed.

We reported a new method to acquire crassicauline A **(3)**, which had not been reported in the literature before. In our research, the semi synthesis of **3** from dehydroyunaconitine **(2)** was one of the most difficult steps. After a series of unsuccessful experiments, we finally found a practicable method. Compared with other methods, this one below was more suitable for production application and more concise; moreover, the cost was lower with higher yield.

In summary, we would continue to study the nature of yunaconitine **(1)** further. Previous studies have shown that (C-3, C-8, C-14) bonds of yunaconitine **(1)** have high pharmacological activity. Hence, we would tentatively synthesize a series of derivatives and establish some drug models in mice firstly; then, we expect to find better analgesic effect and fewer side effects with lower toxic compounds. Generally, this subject would be very useful for studies of structure–function relationship regard to diterpenoid alkaloids.

## Experimental Sections

UV spectra were measured on a UV 210A Shimadzu spectrometer. IR spectra were recorded on a Vector 22 spectrometer with KBr pellets. Optical rotations were measured on Rudolph Autopol VI polarimeter. (Rudolph Research Analytical, Hacketstown, NJ, USA).One-dimensional NMR spectra were recorded with Avance spectrometer operating at 400 MHz for ^1^H and at 100 MHz for ^13^C. The chemical shifts (*δ*) were measured in CDCl_3_ (solvent signals: *δ*_H_ 7.24, *δ*_C_ 76.90) with TMS as an internal standard. ESI mass spectra were recorded on VG Auto Spec-3000 spectrometer.

### Separation of Yunaconitine (**1**)

In a normal method [11], firstly, we crushed the roots of *A. hemsleyanum* or *A. geniculatum* into powders. Secondly, above-mentioned powders (1.5 kg) were soaked by 10% sodium carbonate and extracted with chloroform. Thirdly, the concentrated extracts were diluted with water and acidified with 2% hydrochloric acid. Fourthly, the liquor from last step was filtered; the filter liquor was alkalized with ammonium hydroxide and extracted with ether. Lastly, the ether solvent was dried over anhydrous sodium sulfate and concentrated under vacuum to acquire yunaconitine **(1)** (15.6 g, yield: 1.04%). After the final step, **1** was recrystallized several times by using ether to form crystals.

### Preparation of Dehydroyunaconitine (**2**)

At first, yunaconitine **(1)** (1.07 g) was dissolved in thionyl chloride (15 ml) and the resulting mixture in a round bottom flask was refluxed at 80 ℃ for 11 h. In the second step, the mixture above was filtered and the filter liquor was evaporated to dryness under reduced pressure to give a residue (1.45 g). Thirdly, the residue was dissolved in H_2_O and alkalized to pH 8 with saturated sodium carbonate. After that, the liquor from last step was extracted with dichloromethane and the dichloromethane solvent was dried over anhydrous sodium sulfate. Finally, the above-mentioned solvent was concentrated under vacuum to obtain dehydroyunaconitine **(2)** and the compound was purified with silica gel column chromatography (eluted with acetone: petroleum ether 3:7; chloroform: methanol 9.5:0.5, respectively) to afford the pure product **2**. (836 mg, yield: 80.3%). **2** could be recrystallized by using acetone: n-hexane mixed in specific proportion to form crystals.

Dehydroyunaconitine **(2)**: cubic crystals, mp 271.5–274.0 °C;${[\alpha ]}_{D}^{20.5}$ + 42.65 (*c* 0.068, CHCl_3_); UV (MeOH) λ_max_ (log ε): 201.5 (3.63), 207.5 (3.73), 259 (3.80) nm. One of the IR (KBr) ν_max_ 1635 cm^−1^. The ^13^C NMR spectra (CDCl_3,_ 100 MHz) see Table 1. ESIMS *m/z* 642 [M+H]^+^; positive ion HRESIMS *m/z* 642.3200 (calcd for C_35_H_47_NO_11_ [M+H]^+^, 642.3199).

**Table 1:** ^13^C NMR Data of compounds **1, 2, 3** in CDCl_3_

NO	**1**	**2**	**3**
C(1)	83.1	83.5	85.0
C(2)	33.5	125.3	26.3
C(3)	71.4	137.6	34.9
C(4)	43.1	40.8	39.1
C(5)	47.4	47.8	50.2
C(6)	82.2	80.9	83.8
C(7)	44.7	44.5	45.1
C(8)	85.6	85.8	85.6
C(9)	48.7	46.4	50.0
C(10)	40.8	41.0	41.0
C(11)	50.2	48.8	49.1
C(12)	35.1	33.8	35.8
C(13)	74.6	74.8	74.8
C(14)	78.5	78.5	78.6
C(15)	39.5	40.1	39.3
C(16)	83.5	83.5	83.1
C(17)	61.6	59.2	61.7
C(18)	76.7	78.2	80.4
C(19)	48.7	52.6	53.6
N-CH_2_-CH_3_	47.4	47.8	49.6
N-CH_2_-CH_3_	13.1	12.4	13.4
1-OCH_3_	55.7	56.1	56.0
6-OCH_3_	58.7	58.8	58.7
16-OCH_3_	57.7	57.8	57.7
18-OCH_3_	59.0	59.2	59.0
O = C-CH_3_	169.9	169.8	169.7
O = C-CH_3_	21.5	21.5	21.6
C(7″)	166.1	165.8	166.0
C(1″)	122.5	122.6	122.8
C(2″) or C(6″)	131.6	131.6	131.7
C(3″) or C(5″)	113.7	113.8	113.7
C(4″)	163.5	163.5	163.5
4″-OCH_3_	55.3	55.4	55.4

Chemical shifts (*δ*) in *ppm* relative to TMS in CDCl_3_

### Preparation of Crassicauline A (**3**)

Firstly, dehydroyunaconitine **(2)** (250 mg) was dissolved in 95% ethanol (5 mL) and under hydrogen reduction with Raney Ni catalysis (1.5 g), the resulting mixture in a round bottom flask was stirred at room temperature for 8 h. Next, after reaction was completed, the Raney Ni was removed by filtration. Lastly, the filter liquor was evaporated to dryness under reduced pressure to acquire a product **3** as white powders (238 mg, yield: 94.9%).

**3** had the same data (^1^H NMR, ^13^C NMR, ESIMS) as standard sample of crassicauline A; the ^13^C NMR spectra of **3** see Table 1. At the same time, **3** also showed the same spot as crassicauline A in thin-layer chromatography. Therefore, the product **3** could be proved to be crassicauline A.
